# Integration of genetic, genomic and transcriptomic information identifies putative regulators of adventitious root formation in *Populus*

**DOI:** 10.1186/s12870-016-0753-0

**Published:** 2016-03-16

**Authors:** Cintia L. Ribeiro, Cynthia M. Silva, Derek R. Drost, Evandro Novaes, Carolina R. D. B. Novaes, Christopher Dervinis, Matias Kirst

**Affiliations:** School of Forest Resources and Conservation, University of Florida, P.O. Box 110410, Gainesville, FL 32611 USA; Plant Molecular and Cellular Biology Graduate Program, University of Florida, P.O. Box 110690, Gainesville, FL 32611 USA; University of Florida Genetics Institute, University of Florida, P.O. Box 103610, Gainesville, FL 32611 USA; Present Address: Monsanto Company, 700 Chesterfield Pkwy W, Chesterfield, MO 63017 USA; Present Address: Seminis, Inc., 37437 State Highway 16, Woodland, CA 95695 USA; Present Address: Universidade Federal de Goiás, Av. Esperança s/n°, Goiânia, GO 74001-970 Brazil

**Keywords:** Adventitious root, QTL, Populus, SUR2, Vegetative propagation

## Abstract

**Background:**

Adventitious roots (AR) develop from tissues other than the primary root, in a process physiologically regulated by phytohormones. Adventitious roots provide structural support and contribute to water and nutrient absorption, and are critical for commercial vegetative propagation of several crops. Here we quantified the number of AR, root architectural traits and root biomass in cuttings from a pseudo-backcross population of *Populus deltoides* and *Populus trichocarpa*. Quantitative trait loci (QTL) mapping and whole-transcriptome analysis of individuals with alternative QTL alleles for AR number were used to identify putative regulators of AR development.

**Results:**

Parental individuals and progeny showed extensive segregation for AR developmental traits. Quantitative trait loci for number of AR mapped consistently in the same interval of linkage group (LG) II and LG XIV, explaining 7–10 % of the phenotypic variation. A time series transcriptome analysis identified 26,121 genes differentially expressed during AR development, particularly during the first 24 h after cuttings were harvested. Of those, 1929 genes were differentially regulated between individuals carrying alternative alleles for the two QTL for number of AR, in one or more time point. Eighty-one of these genes were physically located within the QTL intervals for number of AR, including putative homologs of the *Arabidopsis* genes *SUPERROOT2* (*SUR2*) and *TRYPTOPHAN SYNTHASE ALPHA CHAIN (TSA1)*, both of which are involved in the auxin indole-3-acetic acid (IAA) biosynthesis pathway.

**Conclusions:**

This study suggests the involvement of two genes of the tryptophan-dependent auxin biosynthesis pathway, *SUR2* and *TSA1*, in the regulation of a critical trait for the clonal propagation of woody species. A possible model for this regulation is that poplar individuals that have poor AR formation synthesize auxin indole-3-acetic acid (IAA) primarily through the tryptophan (Trp) pathway. Much of the Trp pathway flux appears to be directed to the synthesis of indole glucosinolates (IG), as suggested by the over-expression of *SUR2*. Individuals that are efficient in AR formation may utilize alternative (non-Trp) pathways to synthesize IAA, based on the observation that they down-regulate the expression of *TSA1*, one of the critical steps in the synthesis of tryptophan.

**Electronic supplementary material:**

The online version of this article (doi:10.1186/s12870-016-0753-0) contains supplementary material, which is available to authorized users.

## Background

Adventitious roots (AR) develop from plant tissues other than the primary root, providing structural support and contributing to water and nutrient absorption [1]. Adventitious and lateral roots follow a common developmental program, although dedifferentiation of already committed cells is required for AR formation [2]. Formation of AR occurs in three phases that may overlap: (1) dedifferentiation of previously committed cells (typically secondary phloem cells); (2) induction, when cells begin to divide to form an internal root meristem; and (3) elongation, when the root-primordia grows and emerges from the stem [1]. When ARs are developed from stem cuttings, wound response also occurs, which activates repair responses and systemic signaling cycles [3]. Usually, AR primordia arise close to the phloem and cambium, at the ray cells or in bud or leaf gaps. Adventitious roots may also arise in the pericycle, between the endodermis and phloem in roots [3]. The timing of each phase of AR formation varies among species and depends on external stimuli, but the first root meristems are frequently observed after 96 h [1, 4, 5].

Phytohormones are critical endogenous factors in AR formation, acting directly on cell division and growth, or indirectly, interacting with other molecules or phytohormones [6]. Auxin is the principal phytohormone that initiates rooting and is critical for the first phases of AR development [7], although inhibitory during elongation. Ethylene is likely to interact with auxin to control adventitious rooting in stems or stem cuttings [2], with some studies suggesting that auxin promotes dedifferentiation through stimulation of ethylene synthesis [8]. Although ethylene is promotive during the first phase of dedifferentiation, it is inhibitory during the induction phase [4]. Cytokinins have also been shown to impact AR formation [9], and interact with auxin to form the quiescent center [7]. Gibberellins appear to negatively impact the initial formation of ARs by interfering with the polar transport of auxin [10], while acting positively in their emergence and elongation [11]. Finally, strigolactones have also shown to impact AR formation [12, 13], although the contribution to the phenotype through interactions with other hormones remains to be uncovered. Regardless of the mechanisms of hormonal regulation, the initial development of ARs is primarily controlled by the availability of auxin and its proper localization, while most other hormones act as inhibitors or in combination with auxin.

The general role of phytohormones in AR formation is relatively well known, but few genes implicated in this developmental process have been identified. While the genes and molecular mechanisms that regulate AR in woody perennial species are unknown, AR development is clearly under significant genetic control in woody species (for instance, see [14, 15]). Species and hybrids in the Aigeiros and Tacamahaca sections of the genus *Populus* are among the taxa that are able to produce adventitious roots from cuttings, but considerable variation in the degree and vigor of rooting exists [16]. Therefore, poplar hybrids are particularly suitable for studying the genetic control of AR formation because there is extensive variation for the trait among species, and well-established genetic and genomic resources [17–20]. Most important, the DNA sequence of the *P. trichocarpa* genotype Nisqually-1 [21] and whole-transcriptome microarrays [22] enable the integration of genomic information with the quantitative genetic dissection of complex traits to uncover genes implicated in their variation using genetical genomics [23].

Here we report the genetic dissection of the variation in AR formation between two of the most economically important woody species in North America, *P. deltoides* and *P. trichocarpa*. We demonstrate the integration of traditional quantitative genetic methods with genomic information measured during the developmental program of AR formation, to identify major putative genes and hormonal biosynthesis pathways implicated in the control of this trait in the genus *Populus*.

## Results

### Adventitious rooting in the parental individuals

**Fig. 1:**
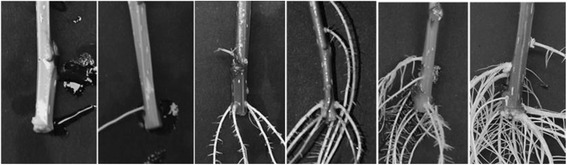
Sample segregation of root number detected observed in family 52–124, after 18 days in hydroponic solution

Adventitious root formation was characterized in a *Populus* pseudo-backcross population, referred hereafter as pedigree 52–124 [24, 25] (Fig. 1). The population was established by crossing a hybrid female parent (*P. trichocarpa* × *P. deltoides*, genotype 52–225) with a pure *P. deltoides* (genotype D124). Cuttings from the parental individuals and 236 individuals from the progeny were placed in a hydroponic solution, and visible ARs were counted daily. Adventitious root primordia were observed after day 5 and over 85 % of the cuttings had developed roots in the 18th day. In both parental individuals, AR formation started almost simultaneously, but the total number of visible roots was significantly lower (*P* < 0.01) in the pure *P. deltoides* male parent until the 17th day, in comparison to the hybrid female parent (Additional file 1). After the 17th day, the difference in the number of roots observed in the two parents was no longer significant. Therefore, both parents appear to have a similar capacity to develop AR, but there is a delayed development in *P. deltoides* relative to the hybrid parent (*P. trichocarpa* × *P. deltoides*).

### Quantitative genetic control of adventitious root formation

The broad-sense heritability (H^2^) was calculated to estimate the extent of AR formation that is genetically controlled in pedigree 52–124, following previous studies that suggested high genetic control for the trait in *Populus* [26–28]. In this study, the heritability for number of roots was moderate (*H*^*2*^ = 0.27–0.34), similar to other complex traits previously analyzed in this pedigree [25].

In addition to root count, several architectural traits were measured in roots harvested after cuttings were maintained 18 days in hydroponic solution. The traits analyzed included total root length, surface area, volume and average diameter of total roots, and number of first order (primary) roots and root branches. All root architectural traits and total dry-weight showed transgressive segregation (Additional file 2). For these architectural traits, the heritability ranged from 0.12 for length of root branches to 0.26 for average diameter (Table 1).

**Table 1:** Broad sense pedigree heritability estimates for adventitious root-related phenotypes measured

	Phenotypic values	Mean ± SD		
Trait	*P. trichocarpa* × *P. deltoides*	*P. deltoides*	Progeny Variation (Mean ± SD)	Broad sense heritability *H* ^*2*^ ± SD
Root architectural traits				
Total root length (cm)	56.5 ± 6.2	86.4 ± 9.0	123.1 ± 94	0.153 ± 0.051
Total root surface area (cm^2^)	10.0 ± 1.1	23.4 ± 2.4	25.1 ± 19	0.169 ± 0.052
Total root volume (cm^3^)	0.14 ± 0.1	0.51 ± 0.1	0.42 ± 0.3	0.185 ± 0.053
Average diameter (mm)	0.56 ± 0.2	0.93 ± 0.1	0.70 ± 0.2	0.236 ± 0.055
Length of root branches (cm)	25.6 ± 10.2	28.5 ± 8.6	55.7 ± 49	0.115 ± 0.049
Surface area of root branches (cm^2^)	2.90 ± 0.3	2.40 ± 0.1	4.33 ± 3.8	0.117 ± 0.049
Volume of root branches (cm^3^)	0.28 ± 0.8	0.20 ± 0.6	0.034 ± 0.03	0.120 ± 0.049
Total length of primary roots (cm)	30.8 ± 9.1	57.6 ± 8.2	67.1 ± 50	0.193 ± 0.053
Surface area of primary roots (cm^2^)	6.90 ± 1.5	20.1 ± 2.5	19.4 ± 15	0.187 ± 0.053
Volume of primary roots (cm^3^)	0.19 ± 0.1	0.61 ± 0.1	0.49 ± 0.4	0.177 ± 0.053
Number of adventitious roots (day 18)	7.36 ± 0.8	4.92 ± 0.5	5.92 ± 4.0	0.341 ± 0.056
Root biomass (mg)	4.73 ± 0.9	27.5 ± 3.2	19.3 ± 20	0.144 ± 0.051

**Table 2:** Putative regulatory motifs in significantly higher frequency among genes belonging to the superroot2 cluster, that are more highly expressed in individuals that inherited the *P. trichocarpa* QTL allele (PtQTL category), based on Fisher’s exact test

Motif Sequence	*p*-value two side test	Annotation
TGACY	0.02854664	ABA responsive element, ABRE3
TTATTT	0.02854664	highly active synthetic auxin response
RTTTTTR	0.01348537	highly active synthetic auxin response
AGATC	0.03372874	ERE (ethylene responsive element)
TTGAC	0.03372874	highly active synthetic auxin response
CCTTTT	0.01817917	highly active synthetic auxin respone
AAAGAT	0.00121528	ERE (ethylene responsive element)
TATTCT	0.01943102	highly active synthetic auxin response
CAANNNNATC	0.02472731	highly active synthetic auxin response
CAACA	0.02854664	ABA responsive element, ABRE3
GATAA	0.02854664	GARE (gibberellic acid responsive element)
RGATY	0.02854664	MeJa-responsive element (MeJaRE)
AATAAT	0.03372874	TATA Box
TATTAAT	0.00474209	TATA Box
AATAAA	0.02854664	TATA Box
ACGTG	0.04506551	ACGT motif” related to root expression
ATATT	0.02854664	RSE (root-specific element)
CANNTG	0.02854664	ACGT motif related to root expression
CAAACAC	0.00985829	RSR (root specific region) S000243
AATTAAA	0.01817917	WAR (wounding activating region)
CWWWWWWWWG	0.04047776	Wound-responsive element (WRE)
ATAGAA	0.01943102	WAR (wounding activating region)
TGHAAARK	0.0418575	Binding site of wound-inducible
ATATTTAWW	0.01082454	Wound-responsive element (WRE)
GGTTAA	0.04506551	Stress responsive element (SRE)
ATGGTA	0.03390743	Stress responsive element (SRE)
GATAAG	0.04970707	Stress responsive element (SRE)
TATTAG	0.0418575	SE2 (stem element 2)

**Fig. 2:**
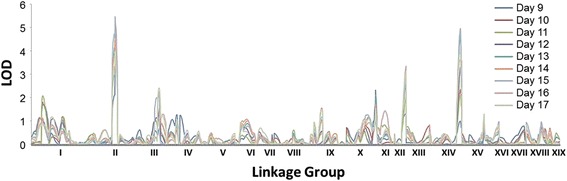
Genome-wide composite interval mapping scan for number of roots detected in family 52–124, 9–17 days after cuttings were placed in hydroponic solution

Quantitative trait loci (QTL) analyses were performed for root architectural traits and for the number of roots counted after 18 days in hydroponic culture, using the genetic map of the hybrid mother [22]. For root architectural traits and root biomass, 15 QTLs were detected on the mother map, and the phenotypic variation explained by each QTL ranged from 6 to 11 % (Additional file 3). Quantitative trait loci for number of ARs mapped consistently in the same intervals of linkage group (LG) II and LG XIV, and explained 7–10 % of the phenotypic variation (Fig. 2 and Additional file 4). The logarithm of odds (LOD) score of these QTL reached 5.60 (QTL in LG II) and 4.99 (QTL in LG XIV). The QTL on LG II spans 34.89 centimorgans (cM) and includes 380 genes, while the QTL on LG XIV span 26.81 cM, with 241 genes. For identification of elements that regulate the number of ARs, further analysis focused on genes located within both QTL intervals on LG II and LG XIV.

### Transcriptome analysis of individuals with alternative alleles at the AR QTL

**Fig. 3:**
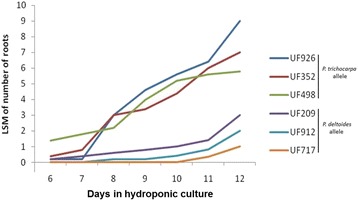
Least square means number of adventitious roots developed on extreme genotypes selected for gene expression analysis

To define putative regulators of AR formation, we searched for transcripts within QTLs on both LG II and XIV that were differentially regulated between individuals carrying the alternative parental alleles. This analysis assumes that genetic differences between individuals that inherited alternative QTL alleles results in differences in gene expression that impact AR – i.e. the trait is at least partially controlled by differences in transcript regulation. Gene expression in three individuals carrying alleles originated from the *P. trichocarpa* grandparent (UF352, UF498 and UF926, referred hereafter as the *Pt*QTL genotype category) was contrasted with those with alleles from the *P. deltoides* grandparent (UF717, UF209 and UF912 or the *Pd*QTL genotype category). These individuals were randomly selected among those that inherited the QTL flanking markers from either the *P. trichocarpa* grandparent (*Pt*QTL genotype category) or the *P. deltoides* grandparent (*Pd*QTL genotype category). For this transcriptome analysis, 25 cuttings of each of the six selected individuals were grown in hydroponic solution, and basal (1 cm) cutting sections from four biological replicates of each individual were collected at each of five time points (0, 24, 48, 96 and 192 h after cuttings were harvested). We emphasized sample collection in the first 96 h because previous studies suggest that AR formation initiates within that period [29]. Five additional biological replicates of each individual were maintained in hydroponic growth conditions until day 12, and confirmed that root development was consistent with the phenotype observed in the QTL detection experiment (Fig. 3). The transcriptome response of cuttings in hydroponic solution was assessed by whole-transcriptome microarrays developed previously [22, 24]. The microarray data generated for each gene was evaluated separately using analysis of variance (ANOVA) with time (0, 24, 48, 96 and 192 h), genotype (UF209, UF352, UF498, UF717, UF912 and UF926) and genotype × time interactions treated as fixed effects (see Methods). For each gene, an ANOVA F-test was carried out to identify if there were significant differences in expression among times of sample collection (0, 24, 48, 96 and 192 h). Significance was determined based on a false discovery rate (FDR) of 5 %. In addition, expression of each gene was compared between the individuals in the *Pd*QTL genotype category (UF717, UF209 and UF912) and the individuals in the *Pt*QTL genotype category (UF352, UF498 and UF926), at each time of sample collection. This analysis was carried out to assess the effect of the AR QTL on gene expression using a 5 % FDR significance threshold, and is referred hereafter as the QTL genotype effect. We focused all further analysis on two comparisons: (1) time effect, and (2) QTL genotype effect.

#### Time effect

The F-test for the effect of the time of sample collection identified 26,121 putative genes as significantly differently expressed (FDR < 5 %) between at least two time points in the experiment (Additional file 5). To define the time at which the most significant changes in gene expression occurred, a comparison of gene expression between consecutive time points (i.e. time points 0–24, 24–48, 48–96 and 96–192 h) was performed (Additional file 6). Most differences in transcript levels occurred during the first 24 h of the experiment (i.e. between 0 and 24 h). Such extensive changes in the transcriptome are likely associated with stress and wounding responses that occurs immediately upon harvest of cuttings and their placement in the hydroponic solution, instead of solely due to the AR development. On the other hand, only ten genes were differentially regulated between 96 and 192 h in hydroponic culture, suggesting that these two time points are within the same rooting phase.

#### QTL genotype effect

Next we sought to identify genes differentially expressed between individuals in the *Pd*QTL and *Pt*QTL categories, at each time point. The aim of this comparison is to identify genes that are differentially regulated during the initial development of AR, between individuals that carry alternative alleles at the QTL that control the trait. Genes differentially regulated between individuals in the *Pd*QTL and *Pt*QTL categories, at any of the time points, and located within the QTL intervals, represent candidate AR regulators. We identified 1929 genes differentially regulated between genotypes in the *Pt*QTL and *Pd*QTL categories, in at least one of the time points of tissue collection (Additional file 7). Of these 1929 genes, 81 are located within the QTL intervals on LG II and XIV. Among these, two putative homologues of genes that encode for enzymes of the tryptophan biosynthesis pathway are of particular interest: POPTR_0002s04640 and POPTR_0002s02770. Tryptophan is a precursor of auxin [30, 31], the primary hormone regulator of AR formation. The gene POPTR_0002s04640 is a putative homolog of *TSA1* (*TRYPTOPHAN SYNTHASE ALPHA CHAIN*). The enzyme encoded by *TSA1* catalyzes the conversion of indole-3-glycerolphosphate to indole, the second to last reaction in the tryptophan biosynthesis, and showed lower expression among the better rooting individuals of the *Pt*QTL category at 48 and 192 h. POPTR_0002s02770, the putative homolog of the *Arabidopsis* gene *SUR2* (*SUPERROOT2*), was significantly more highly expressed among individuals in the *Pd*QTL category, compared to those in *Pt*QTL, at 192 h. A *SUR2* knockout mutant has been shown to cause auxin overproduction and an abnormally high number of adventitious roots [32, 33]. In the analysis of the time effect, POPTR_0002s02770 was also significantly differentially expressed between times 0 and 24 h, among individuals in both the *Pd*QTL and *Pt*QTL categories.

Other genes implicated in phytohormone response and located within the target QTL intervals were also differentially regulated between individuals in the *Pd*QTL and *Pt*QTL categories, at several time points. For instance, at 96 and 192 h, higher expression was observed for the gene POPTR_0002s02420, which is a homolog of *Arabidopsis GA-STIMULATED TRANSCRIPT 1 (GASA1)* among individuals of the *Pd*QTL category. GASA1 is involved in response to gibberellins stimulus, brassinosteroid, abscisic acid stimulus and unidimensional cell growth [34]. At 48 and 96 h, a homolog of the *Arabidopsis* gene *ETO1-LIKE PROTEIN 1 (EOL1)*, POPTR_0002s04910, was more highly expressed in individuals of the *Pt*QTL category. *EOL1* encodes a paralog of *ETHYLENE-OVERPRODUCER1*, which is a negative regulator of the gene *1-AMINOCYCLOPROPANE-1-CARBOXYLATE SYNTHASE 5*, a key enzyme in ethylene biosynthesis pathway [35].

### Clustering the difference in transcriptome response of *Pt*QTL and *Pd*QTL genotypes

Genes differentially regulated between individuals in the *Pt*QTL and *Pd*QTL categories are likely to be part of an orchestrated response that distinguishes the two species in their ability to form AR. To uncover the differential functional responses, we clustered the 1929 genes differentially regulated between the *Pt*QTL and *Pd*QTL categories, based on the difference in transcript abundance between the two at each time point. Genes with a common pattern of differential regulation throughout the experiment were clustered using a Modulated Modularity Clustering (MMC) graph-based method [36]. Sixty clusters were identified, varying from 2 to 148 transcripts in size, and eight genes remained unclustered (Additional file 8 and Additional file 9).

### Transcription factor binding site analysis

Of all the genes identified as differentially regulated between individuals in the *Pd*QTL and *Pt*QTL categories, and located within the QTL intervals, only POPTR_0002s02770 is a homolog to an *Arabidopsis* gene previously shown to control AR development (*SUPERROOT2,* Boerjan, 1995 [32]; Delarue et al., 1998 [33]). Because of the potential role of POPTR_0002s02770 in AR formation of poplars, we used the PLACE (plant cis-acting regulatory DNA elements) database to identify conserved motifs over represented in the cluster containing this gene. This analysis is constrained by the fact that it is solely based on motifs detected in *P. trichocarpa*, because a suitable reference genome sequence is not available for *P. deltoides*. A total of 48 genes grouped in the superroot2 cluster, and half of them were highly expressed in individuals of the *Pd*QTL category while the other half were highly expressed in individuals of the *Pt*QTL category. We hypothesized that genes in this cluster would share motifs related to hormone regulation, particularly auxin. A Fisher’s exact test was performed to identify motifs in higher frequency in one of the two QTL categories, which identified 25 significant motifs (Table 2). Interestingly, all motifs were significantly (*P*-value > 0.05) enriched in the *Pt*QTL category group of genes. Half of the motifs detected have been previously described to be associated with phytohormonal response, mainly auxin (6 motifs categories) but also ethylene, abscisic acid and gibberellins. Also, several motifs are directly related to rooting and wounding response. These results add evidence to the influence of these co-expressed genes in regulating adventitious root formation.

## Discussion and conclusions

The capacity of plants to develop AR is extensively used by many industries and research segments to propagate elite individuals selected in breeding programs or in natural populations. Significant economic losses are associated with cuttings producing poor quality root systems or complete failing to form them [1]. In this study we combined the genetic (QTL) analysis of a segregating population, with genome and transcriptome data to identify putative regulators of AR development in *Populus*. The analysis focused on the segregation of alleles from a hybrid of *P. trichocarpa* and *P. deltoides* in a mapping population. Previous observations identified the two species as being contrastingly distinct with respect to AR formation.

We detected a moderate heritability for most AR developmental traits analyzed, in line with similar studies in other *Populus* species [37, 38]. Only two QTL studies on AR development in *Populus* had been previously reported [26, 39]. Han and colleagues studied the quantitative genetic aspect of in vitro adventitious root formation and shoot regeneration, and Zhang et al. [26] used functional mapping to detect QTLs for number of roots and maximum root length measured at different time points. However, no common QTL were detected between those studies and the results reported here. Differences might be attributed to the use of families with distinct genetic backgrounds, growth conditions and type of cuttings.

The transcriptome data indicates that the largest number of genes is differentially regulated in the first 24 h after cuttings were harvested, regardless of the QTL allele inherited. This result is expected to be due to hormonal and gene regulation changes related to wound response and de-differentiation of the cells to a meristematic state capable of cell division. Expectedly, numerous genes up-regulated during the first 24 h reflect these changes. These includes *CPC902* (*CONDENSIN COMPLEX COMPONENTS SUBUNIT C*), a homolog of the Arabidopsis gene *SMC1* (*STRUCTURAL MAINTENANCE OF CHROMOSOMES 1*). *SMC1* encodes for one of the proteins of the cohesion complex family [40], necessary for correct chromosome segregation during nuclear divisions, possibly indicating the initiation of cell divisions necessary for root meristem organization.

**Fig. 4:**
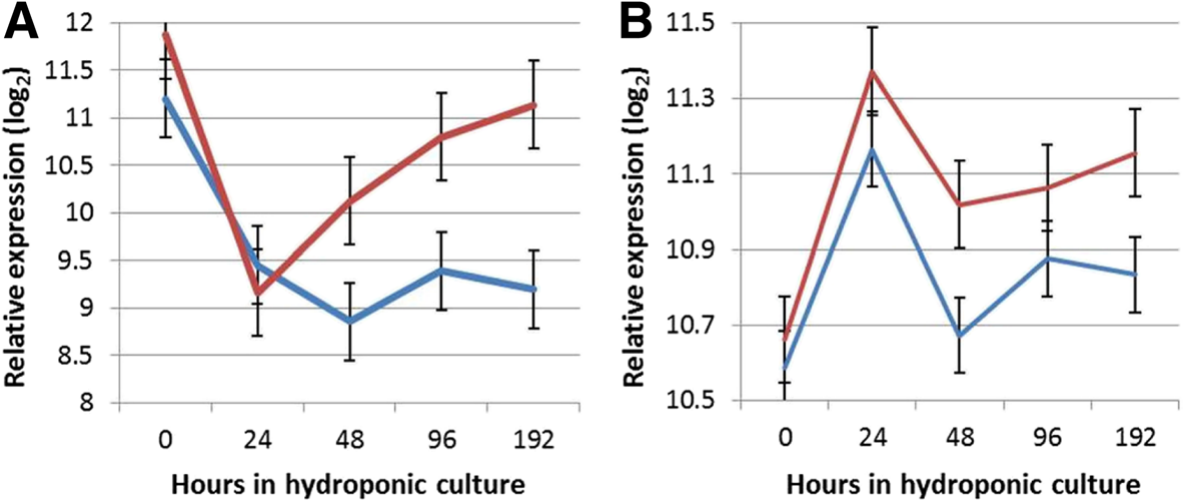
Relative expression level (log2) of *SUR2* (Panel **a**) and *TSA1* (Panel **b**) at different time points, measured as the least square mean of individuals in the *Pt*QTL category (*blue line*) and the *Pd*QTL category (*red line*). Error bars show the standard error

The identification of transcripts differentially regulated at different time points following the collecting of cuttings provides a broad, transcriptome overview of genes and pathways that may participate in wounding and cell de-differentiation, root induction and elongation [1]. However, it does not identify a defined set of genes or specific polymorphisms that are responsible for the phenotypic differences between AR formation in *P. deltoides* and *P. trichocarpa*. To achieve this goal we contrasted gene expression between individuals carrying alternative alleles that control AR formation, detected based on a QTL analysis, at each time point. Genes differentially regulated between individuals that inherited the alternative *P. deltoides* or *P. trichocarpa* QTL alleles were then evaluated for their position in the *Populus* genome, to detect those located within the QTL intervals. Among those genes, *SUR2* undergoes a highly significant reduction in expression between the time the cuttings were taken and the first 24 h in hydroponic culture. This reduction in expression is independent on the genotype at the QTLs—it is observed in those individuals in the *Pt*QTL and *Pd*QTL categories. However, in the following time points (48–192 h), the levels of *SUR2* remain low in the individuals that form AR early (*Pt*QTL category) but rise steeply towards levels detected at 0 h in the poor AR developing individuals (*Pd*QTL category) (Fig. 4a). Interestingly, poplar’s putative homologue of *TSA1,* which encodes for the enzyme that catalyzes the conversion of indole-3-glycerolphosphate to indole, the second to last reaction in the tryptophan biosynthesis, is also located in the LG II QTL interval. *TSA1* also shows lower expression among the better rooting individuals of the *Pt*QTL category in later time points of 48 and 192 h (Fig. 4b). Taken together, the data suggests that poplar genotypes that are limited in AR formation could synthesize auxin indole-3-acetic acid (IAA) primarily through the tryptophan (Trp) pathway. However, much of the pathway flux appears directed towards synthesis of indole glucosinolates (IG) because on the over-expression of *SUR2*. On the contrary, genotypes that are efficient in AR formation down-regulate the synthesis of Trp (by down-regulating *TSA1*) and/or the diversion of the pathway towards synthesis of IG. The auxin IAA has long been postulated to be synthesized through multiple pathways [41], including a Trp-independent pathway [42]. Recently, an *Arabidopsis* indole synthase mutant defective in the Trp-independent auxin biosynthetic pathway was uncovered [43]. Gene expression in the poplar putative homologue was investigated in this study, but showed no significant difference in transcript levels among genoytpes, and over time.

Clearly, gene expression may not reflect protein level changes or other process (such as protein modifications) that could impact IAA biosynthesis. Other genes differentially regulated between individuals in the *Pt*QTL and *Pd*QTL categories, and located within the AR QTL interval, may also be relevant and should be considered upon further analysis. Furthermore, although significant differences in gene expression were detected in *SUR2* and other genes related to IAA biosynthesis, the biological impact of these changes can only be assessed by further experimentation that is beyond the scope of the research described here. Small differences in gene expression may be significant statistically, but have no or limited biological impact. Despite these concerns, this study raises an attractive hypothesis that the difference between AR formation in *P. deltoides* and *P. trichocarpa* is driven by difference in the expression of genes in the IAA biosynthesis pathway, possibly under the control of the poplar homologues of *SUR2* and *TSA1*.

## Methods

### Plant material and phenotypic measurements

The pedigree (52–124) used in this study is a pseudo-backcross between the hybrid female parent 52–225 [*P. trichocarpa* (clone 93–968) × *P. deltoides* (clone ILL-101)] and the unrelated male parent D124 (*P. deltoides*), established by the Natural Resources Research Institute of the University of Minnesota. The parent D124 is from northern Minnesota. The *P. trichocarpa* parent of the hybrid came from western Washington, whereas the *P. deltoides* parent material originated in Illinois. Twelve centimeters (cm) apical cuttings were collected from 234 individuals of pedigree 52–124, as well as the parental individuals. Cuttings were placed in 58 × 41 × 15 cm containers, with up to 59 cuttings per container, and maintained in hydroponic culture (H_2_O buffered at pH 5.7, with 0.5 g L^−1^ of MES) for the duration of the experiments. The experimental design was an incomplete block design with four blocks and three replications, for a total of 708 cuttings. Root emergence was recorded daily at the same time (10 am), until the 18th day of culture. After 18 days in hydroponic solution, roots were harvested, scanned using Scanner CanoScan LiDE 600 F (Cannon) and dried for measurement of total dry-weight. The scanned roots were analyzed using WinRHIZO Pro (Regent Instruments Inc.) for total root length, surface area, volume and average diameter of total roots, first order (primary) roots and root branches. Because the experiment was conducted in a closed environment and all material was destroyed upon completion of measurements, permits are not required by the existing legislation. A public collection established at the University of Florida provides permanent access to the material utilized in this study.

### Statistical analysis

Covariance parameters were estimated for all traits using PROC MIXED (SAS Institute Inc. 9.2® 2004, Cary, NC, USA), considering all variables random in the following model:

$$ {\gamma}_{ijkl}=\mu +{\alpha}_i+{\beta}_j+{\gamma}_{k(j)}+{e}_{ijkl} $$

where *γ*_*ijkl*_ is the phenotypic value of the *i*th genotype in the *j*th block within the *k*th replication, μ is the overall mean, *α*_*i*_ is the random effect of the genotype; *β*_*j*_ is the random effect of replication, *γ*_*k*(*j*)_ is the random effect of incomplete block (within replication) and *e*_*ijkl*_ is the residual error.

Broad-sense pedigree heritability was calculated using the covariance parameter estimates in the following formula:

$$ {H}^2=\frac{\sigma_c^2}{\sigma_c^2+{\sigma}_e^2} $$

where *σ*_*c*_^2^ and *σ*_*e*_^2^ are the variance components corresponding to genotype and residual effect across the three replications, respectively.

A log transformation was applied to all traits, except for number of roots. Least-square means used in the QTL analysis were calculated by including clone as a fixed effect in the model, using PROC MIXED.

### QTL analysis

QTLs for root-related traits were identified based on a linkage map previously described [22, 25]. The linkage map consists of 181 markers chosen on the basis of homogenous distribution in the hybrid female parent. The map had an average density of one marker every 16 cM. QTLs were identified using composite interval mapping [44] in Windows QTL Cartographer v.2.5 using standard model 6 with walk speed of 2 cM. A genome-wide significance level of *P* < 0.05 was established based on 1000 permutations [45].

### Selection of individuals with alternative alleles in QTL regions

Quantitative trait loci for the number of roots were consistently mapped on LG II and XIV (see Results). We classified each individual depending on the allele (*P. trichocarpa* or *P. deltoides*) that was observed in both QTL regions. Four categories were defined: (1) individuals carrying *P. deltoides* or (2) *P. trichocarpa* alleles at both QTLs, and (3) individuals carrying *P. trichocarpa* alleles at the QTL in LGII and *P. deltoides* alleles in QTL on LG XIV, and (4) vice-versa. Individuals with recombination between markers flanking in each of the two QTL were not grouped into any of the categories. As expected, individuals carrying *P. trichocarpa* alleles in both QTL regions (*Pt*QTL category) generally had more roots than those carrying the *P. deltoides* alleles in those intervals (*Pd*QTL category). For these six individuals we collected 12 cm-long cuttings and established them in the same hydroponic conditions used previously in the QTL detection experiment. The number of new roots formed in these individuals was recorded daily for 12 days, and samples were collected for transcriptome analysis.

### Tissue sampling for microarray analysis

To measure gene expression during adventitious root formation in the three selected individuals from each QTL category (*Pt*QTL and *Pd*QTL), a 1 cm section, measured from the base of each cutting, was collected at 0, 24, 48, 96 and 192 h after placing them in the hydroponic solution. Samples were flash-frozen in liquid nitrogen for posterior RNA extraction. Four biological replicates were collected from each individual, at each time point. In addition, five biological replicates of each individual were maintained in hydroponic growth conditions until day 12 to verify that the root development was consistent with the phenotype observed in the QTL detection experiment.

### RNA extraction, cDNA synthesis and labeling

Total RNA was extracted [46] from the bottom 1 cm stem section collected from each sample. The sample included xylem, phloem and bark. RNA was purified using RNeasy Mini Kit columns (Qiagen), and DNase treated with RNase-Free DNase set (Qiagen). RNA quality was evaluated in 1 % w/v agarose gels. RNA was amplified and cRNA synthesized and labeled using Two Dyes Agilent Low Input Quick Amp Labeling Kit (Agilent). The microarray platform used consisted of single 60-mer probes designed for each of 43,803 annotated gene models from the sequenced genome of *P. trichocarpa* (National Center for Biotechnology Information Gene Expression Omnibus Platform GPL20736). These probes were previously selected for being suitable for analysis of gene expression in this mapping population [22].

### Microarray experimental design and data analysis

A total of 60 microarrays were used in the transcriptome analysis. Gene expression of each of six individuals was analyzed in five time points (0, 24, 48, 96 and 192 h), with four biological replicates per individual and time point. The design was selected to favor contrasting gene expression of samples from different QTL categories (*Pt*QTL and *Pd*QTL) at each time point, as well as samples from the same individual collected from different time points. Data is stored in the National Center for Biotechnology Information Gene Expression Omnibus Series GSE71630. Median values of signal intensities were quantile normalized [47] and log_2_ transformed. Normalized signals were analyzed in SAS 9.2 (SAS Institute Inc. 9.2® 2004, Cary, NC, USA) using a mixed-model ANOVA with genotype and genotype × time interactions as fixed effects, and microarray as random effect. Differences in expression between the group of individuals from the *Pt*QTL and *Pd*QTL categories were estimated at each time point, and the significance was determined based on a false discovery rate (FDR) of 5 % [48]. Genes showing a similar pattern of expression differences between individuals from the *Pt*QTL and *Pd*QTL categories, at all time points, were clustered using a Modulated Modularity Clustering graph-based technique using Spearman correlation [36].

### Annotation

*Populus* gene model transcript sequences were annotated by searching for sequence similarities using BLASTx against *Populus* (JGI v.1.1 and v2.2) and The *Arabidopsis* Information Resource (TAIR v8.0) gene models.

### Transcription factor binding sites analysis

Promoter sequences upstream of the start codon of *P. trichocarpa* gene models were previously extracted [24] to identify presence and absence of common plant *cis*-acting elements. The cluster containing gene POPTR_0002s02770, *Arabidopsis* homolog of SUR2 was divided into two groups based on the gene expression pattern i.e., genes being more highly expressed in the PtQTL or *Pd*QTL category. PLACE (plant *cis*-acting regulatory DNA elements) database of nucleotide motifs [49] was used to identify conserved motifs being over represented in each of these groups and also infer functional roles in coregulated genes. Two-sided fisher exact test was performed in SAS (SAS Institute Inc. 9.2® 2004, Cary, NC, USA) using PROC FREQ to test over-representation of a specific motif in genes being highly expressed among individuals of the *Pd*QTL category against genes highly expressed among individuals of the *Pt*QTL category within SUR2 cluster.

## Availability of supporting data

The microarray data is publically available in the National Center for Biotechnology Information Gene Expression Omnibus under the accession numbers GSE71630.

## Additional files

Additional file 1: Cumulative number of roots formed in the parents of pedigree 52–124. Least-square means of number of adventitious roots developed on the female hybrid parent *Populus trichocarpa* × *P. deltoides* 52*–*225 (red line), and the unrelated male parent *P. deltoides* D124 (blue line)*,* maintained in hydroponic solution for 25 days. (DOCX 67 kb) Additional file 2: Frequency distribution of root architectural and biomass traits. Frequency distribution of least-square means of root architectural and biomass traits measured on 225 individuals of pedigree 52–124. Parents ‘P. deltoides’ (D) and (*P. trichocarpa* × *P.deltoides*) × *P.deltoides* (TD) are indicated. Measurements were made after 18 days of growth in hydroponic solution. Traits are total root length (cm, panel A), total root surface area (cm^2^, panel B), total root volume (cm^3^, panel C), average diameter (mm, panel D), length of root branches (cm, panel E), surface area of root branches (cm^2^, panel F), volume of root branches (cm^3^, panel G), total length of primary roots (cm, panel H), surface area of primary roots (cm^2^, panel I), volume of primary roots (cm^3^, panel J), number of adventitious roots at 18 days (panel K) and root biomass (mg, panel L). (DOCX 326 kb) Additional file 3: QTL detected for root architecture traits and root biomass. Phenotypic variance explained by each QTL interval detected for root architecture traits and root biomass. Respective linkage group (LG), flanking marker location, LOD score and origin of positive allele. (DOCX 17 kb) Additional file 4: QTL detected for the trait number of adventitious roots. Phenotypic variance explained by each QTL interval identified for number of root traits. Respective linkage group (LG), flanking markers location, LOD peak and origin of positive allele. (DOCX 16 kb) Additional file 5: Genes differentially regulated among time point of sample collection. Genes differentially expressed among times of sample collection. The table describes the name of each poplar gene based on all three main annotations of the poplar genome (v. 1.1, v. 2.2 and v. 3.0), the name of the probe on the microarray, the FDR adjusted p-value for the F-test of the effect of TIME in the ANOVA, and the estimate of the effect of TIME at 0, 24, 48, 96 and 192 h. (XLSX 3466 kb) Additional file 6: Differentially regulated genes between time points. Number of genes differentially expressed when contrasting consecutive time points. (DOCX 44 kb) Additional file 7: Differentially regulated genes between *Pd*QTL and *Pt*QTL categories, at each time point. The table describes the name of each poplar gene based on all three main annotations of the genome (v. 1.1, v. 2.2 and v. 3.0), the name of the probe on the microarray, and the gene expression difference (log_2_) between individual in the *Pd*QTL and *Pt*QTL categories. Data is presented only for those genes and time points were the difference in expression between *Pd*QTL and *Pt*QTL was significant for an FDR adjusted p-value of 5 %. (XLSX 139 kb) Additional file 8: Clustering the difference in transcriptome response of *Pt*QTL and *Pd*QTL genotypes. Modulated Modularity Clustering of genes displaying a similar pattern of expression differences between genotypes from the *Pt*QTL and *Pd*QTL categories, at all time points. (DOCX 25 kb) Additional file 9: Gene membership of Modulated Modularity Clusters. Genes membership of Modulated Modularity Clusters detected based on genes displaying a similar pattern of expression differences between genotypes from the *Pt*QTL and *Pd*QTL categories, at all time points. (XLSX 262 kb)

## Abbreviations

AR: adventitious roots
CIM: composite interval mapping
cM: centimorgan
FDR: false-discovery rate
IAA: indole-3-acetic acid
LG: linkage group
LOD: logarithm of the odds
MMC: modulated modularity clustering
QTL: quantitative trait locus
SUR2: SUPERROOT2
Trp: tryptophan
TSA1: TRYPTOPHAN SYNTHASE ALPHA CHAIN
