# Supplementary material for: Integration of genetic, genomic and transcriptomic information identifies putative regulators of adventitious root formation in Populus
Source: BMC Plant Biol. 2016 Mar 16;16:66. doi: 10.1186/s12870-016-0753-0 (PMC4793515; doi:10.1186/s12870-016-0753-0)
Supplement: Additional file 1: — Cumulative number of roots formed in the parents of pedigree 52–124. Least-square means of number of adventitious roots developed on the female hybrid parent Populus trichocarpa × P. deltoides 52–225 (red line), and the unrelated male parent P. deltoides D124 (blue line), maintained in hydroponic solution for 25 days. (DOCX 67 kb) [file 12870_2016_753_MOESM1_ESM.docx]

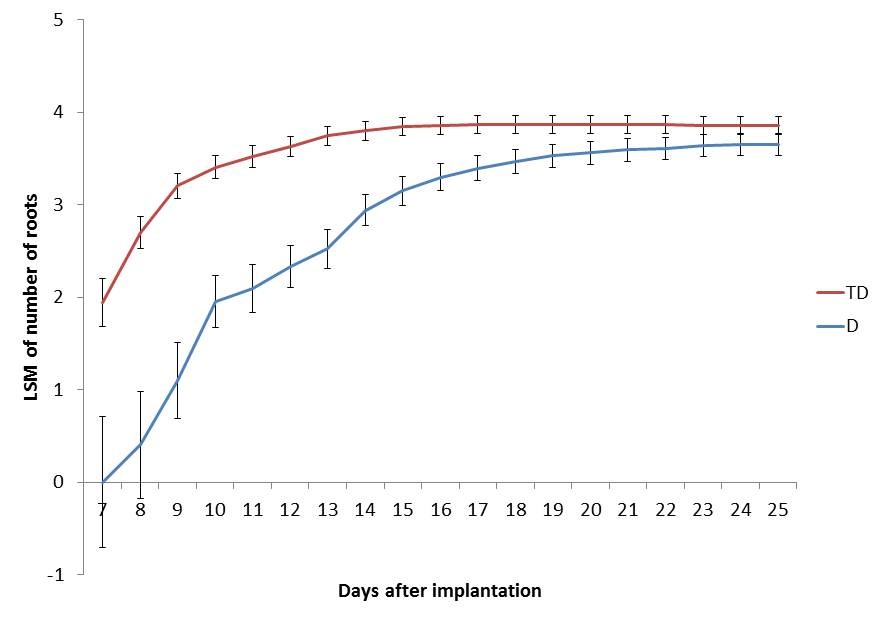


**Days in hydroponic culture**

**Additional file 1.** Cumulative number of roots formed in the parents of family 52-124. Least-square means of number of adventitious roots developed on the female hybrid parent *Populus trichocarpa* × *P. deltoides* 52-225 (red line), and the unrelated male parent *P. deltoides* D124 (blue line)*,* maintained in hydroponic solution for 25 days. Error bars show the standard error. Parents means are significantly diferent (p<0.01) from day 7 to day 17.
