# Supplementary material for: Integration of genetic, genomic and transcriptomic information identifies putative regulators of adventitious root formation in Populus
Source: BMC Plant Biol. 2016 Mar 16;16:66. doi: 10.1186/s12870-016-0753-0 (PMC4793515; doi:10.1186/s12870-016-0753-0)
Supplement: Additional file 2: — Frequency distribution of root architectural and biomass traits. Frequency distribution of least-square means of root architectural and biomass traits measured on 225 individuals of pedigree 52–124. Parents ‘P. deltoides’ (D) and (P. trichocarpa × P.deltoides) × P.deltoides (TD) are indicated. Measurements were made after 18 days of growth in hydroponic solution. Traits are total root length (cm, panel A), total root surface area (cm2, panel B), total root volume (cm3, panel C), average diameter (mm, panel D), length of root branches (cm, panel E), surface area of root branches (cm2, panel F), volume of root branches (cm3, panel G), total length of primary roots (cm, panel H), surface area of primary roots (cm2, panel I), volume of primary roots (cm3, panel J), number of adventitious roots at 18 days (panel K) and root biomass (mg, panel L). (DOCX 326 kb) [file 12870_2016_753_MOESM2_ESM.docx]

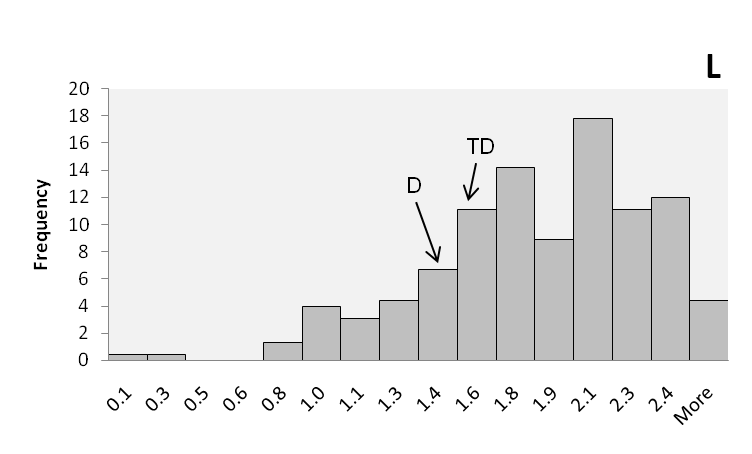

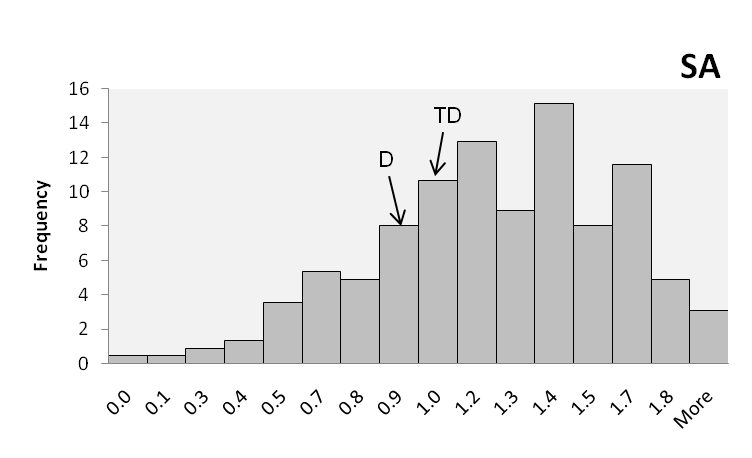


**B**

**A**


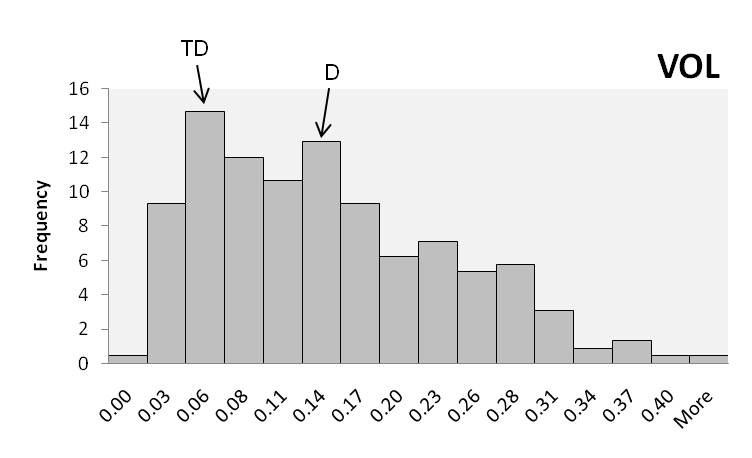

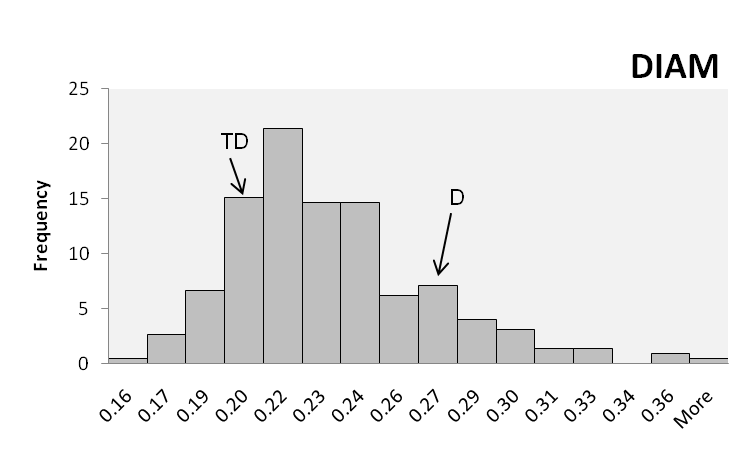


**D**

**C**


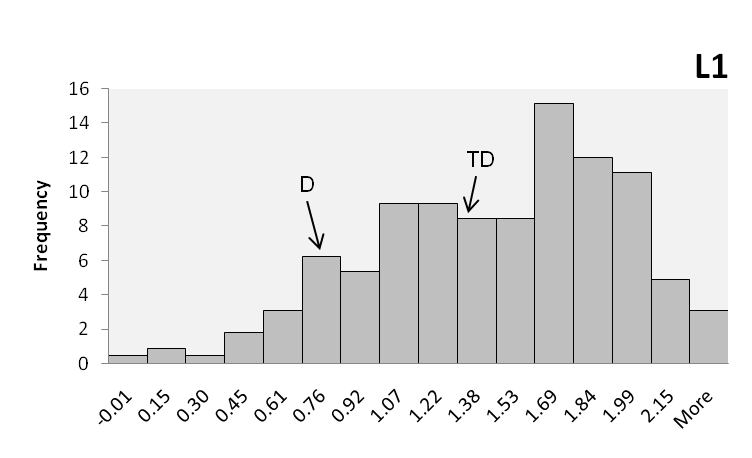

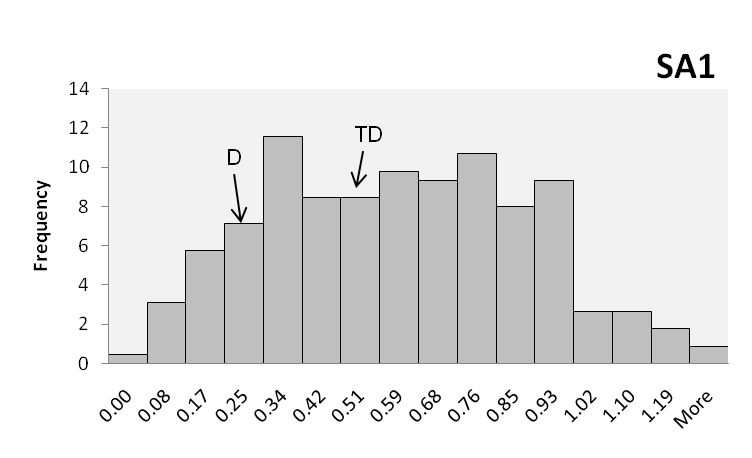


**F**

**E**


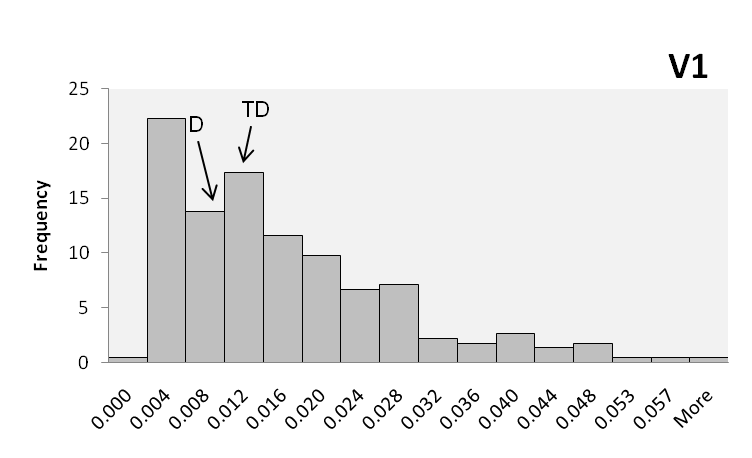

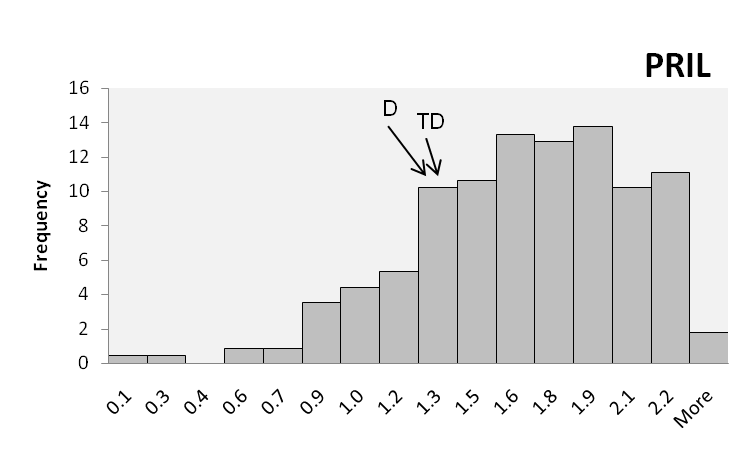


**J**

**H**

**G**


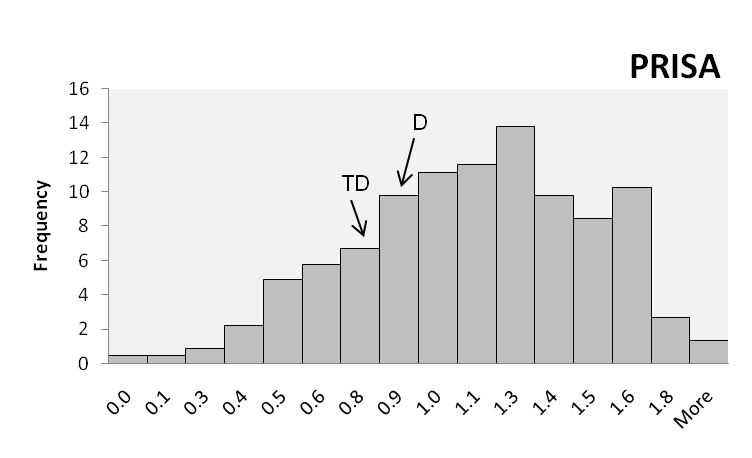

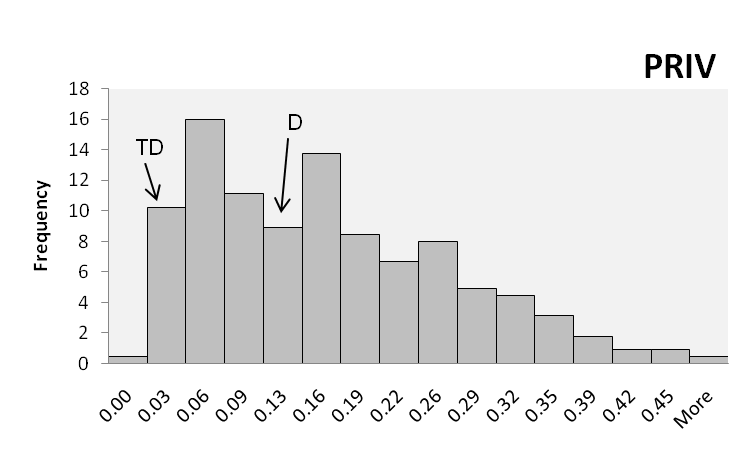


**I**


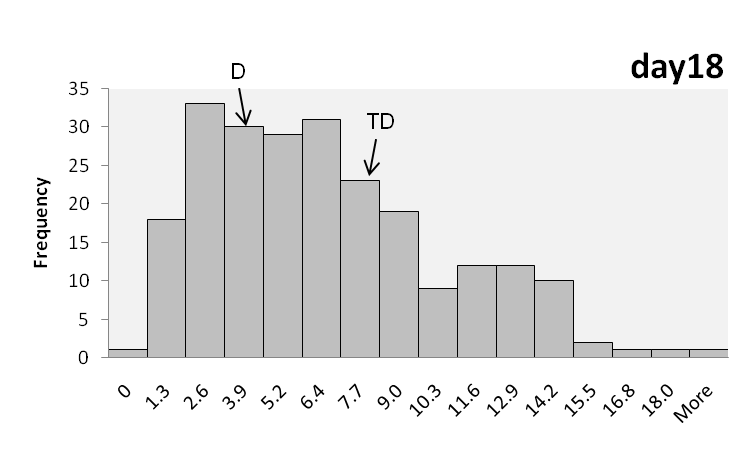

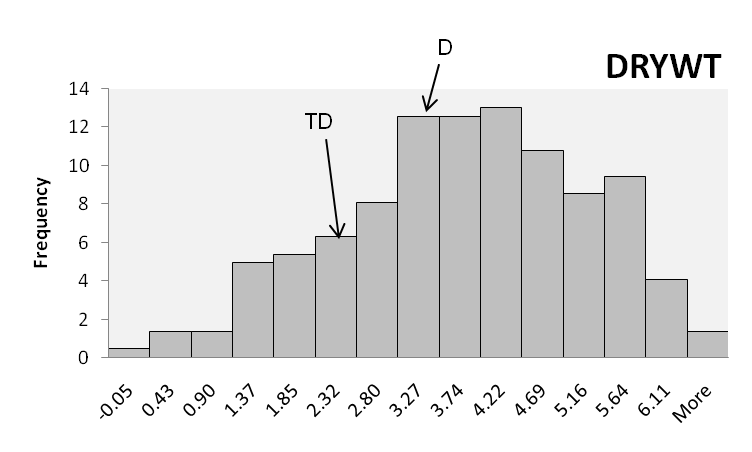


**L**

**K**

**Additional file 2.** Frequency distribution of least-square means of root architectural and biomass traits of 225 individuals of pedigree 52-124. Parents ‘*P. deltoides*’ (D) and (*P. trichocarpa* × *P.deltoides*) × *P.deltoides* (TD) are indicated by arrows. Measurements were made after 18 days of growth in hydroponic solution. Traits are total root length (cm, panel A), total root surface area (cm^2^, panel B), total root volume (cm^3^, panel C), average diameter (mm, panel D), length of root branches (cm, panel E), surface area of root branches (cm^2^, panel F), volume of root branches (cm^3^, panel G), total length of primary roots (cm, panel H), surface area of primary roots (cm^2^, panel I), volume of primary roots (cm^3^, panel J), number of adventitious roots at 18 days (panel K) and root biomass (mg, panel L).
