# Supplementary material for: Integration of genetic, genomic and transcriptomic information identifies putative regulators of adventitious root formation in Populus
Source: BMC Plant Biol. 2016 Mar 16;16:66. doi: 10.1186/s12870-016-0753-0 (PMC4793515; doi:10.1186/s12870-016-0753-0)
Supplement: Additional file 3: — QTL detected for root architecture traits and root biomass. Phenotypic variance explained by each QTL interval detected for root architecture traits and root biomass. Respective linkage group (LG), flanking marker location, LOD score and origin of positive allele. (DOCX 17 kb) [file 12870_2016_753_MOESM3_ESM.docx]

**Additional file 3.** Phenotypic variance explained by each QTL detected for root architecture traits and root biomass. Respective linkage group (LG), flanking marker location, LOD score and origin of positive allele.

|  |  |  |  |  | | | **Flanking markers** | | | |  |  | |  | |
| --- | --- | --- | --- | --- | --- | --- | --- | --- | --- | --- | --- | --- | --- | --- | --- |
| **QTL** | **Trait acronym** | **Trait name** | | | **LG** | **Marker 1** | | **Marker 2** | **LOD peak** | **Origin of positive allele** | | | **Phenotypic variance explained (%)** | |  |
| 1 | L1 | Length of root branches | | | XII | G2643 | | G2673 | 3.80 | *P. deltoides* | | | 7.64 | |  |
| 2 | L | Total root length | | | XII | G2643 | | G2673 | 3.86 | *P. deltoides* | | | 7.18 | |  |
| 3 | L | Total root length | | | XIV | rO386a | | P2515 | 4.30 | *P. deltoides* | | | 10.0 | |  |
| 4 | PRIL | Total length of primary roots | | | II | S96 | | O461 | 3.16 | *P. deltoides* | | | 5.8 | |  |
| 5 | PRIL | Total length of primary roots | | | XII | G2643 | | G674 | 4.45 | *P. deltoides* | | | 8.12 | |  |
| 6 | PRIL | Total length of primary roots | | | XIV | rO386a | | G674 | 5.12 | *P. deltoides* | | | 10.87 | |  |
| 7 | PRISA | Surface area of primary roots | | | XII | G2643 | | G2673 | 4.56 | *P. deltoides* | | | 8.54 | |  |
| 8 | PRISA | Surface area of primary roots | | | XIV | rO386a | | G674 | 4.84 | *P. deltoides* | | | 10.84 | |  |
| 9 | PRIV | Volume of primary roots | | | XII | G2643 | | G2673 | 3.17 | *P. deltoides* | | | 6.12 | |  |
| 10 | PRIV | Volume of primary roots | | | XIV | rO386a | | P2515 | 3.18 | *P. deltoides* | | | 6.76 | |  |
| 11 | DRYWT | Dry Biomass | | | XVII | rG880 | | P648 | 3.08 | *P. trichocarpa* | | | 7.04 | |  |
| 12 | SA | Total root surface area | | | XII | G2643 | | G2673 | 3.95 | *P. deltoides* | | | 7.40 | |  |
| 13 | SA | Total root surface area | | | XIV | rO386a | | G674 | 4.64 | *P. deltoides* | | | 10.03 | |  |
| 14 | VOL | Total root volume | | | XII | G2643 | | G2673 | 3.61 | *P. deltoides* | | | 6.92 | |  |
| 15 | VOL | Total root volume | | | XIV | rO386a | | P2515 | 3.43 | *P. deltoides* | | | 7.22 | |  |
