# Supplementary material for: Integration of genetic, genomic and transcriptomic information identifies putative regulators of adventitious root formation in Populus
Source: BMC Plant Biol. 2016 Mar 16;16:66. doi: 10.1186/s12870-016-0753-0 (PMC4793515; doi:10.1186/s12870-016-0753-0)
Supplement: Additional file 4: — QTL detected for the trait number of adventitious roots. Phenotypic variance explained by each QTL interval identified for number of root traits. Respective linkage group (LG), flanking markers location, LOD peak and origin of positive allele. (DOCX 16 kb) [file 12870_2016_753_MOESM4_ESM.docx]

**Additional file 4.** Phenotypic variance explained by each QTL interval identified for the number of root trait. Respective linkage group (LG), flanking markers location, LOD peak and origin of positive allele.

|  |  |  | **Flanking Markers** | |  |  |  |
| --- | --- | --- | --- | --- | --- | --- | --- |
| **QTL** | **Trait acronym** | **LG** | **Marker 1** | **Marker 2** | **LOD peak** | **Origin of positive allele** | **Phenotypic variance explained (%)** |
| 1 | day9 | II | G734 | rG876 | 5.34 | *P. deltoides* | 10.12 |
| 2 | day9 | IV | O349 | G961 | 4.22 | *P. trichocarpa* | 7.94 |
| 3 | day11 | II | S96 | rG876 | 5.48 | *P. deltoides* | 10.34 |
| 4 | day14 | II | S96 | rG876 | 5.60 | *P. deltoides* | 10.66 |
| 5 | day14 | XIV | rO386a | G674 | 4.99 | *P. deltoides* | 9.67 |
| 6 | day15 | II | S96 | rG876 | 5.00 | *P. deltoides* | 9.33 |
| 7 | day15 | XIV | rO386a | G674 | 5.03 | *P. deltoides* | 8.93 |
| 8 | day16 | II | S96 | O461 | 3.72 | *P. deltoides* | 7.20 |
| 9 | day16 | XIV | rO386a | P2515 | 3.87 | *P. deltoides* | 7.86 |
| 10 | day17 | II | S96 | rG876 | 4.75 | *P. deltoides* | 9.19 |
| 11 | day17 | XIV | rO386a | P2515 | 3.63 | *P. deltoides* | 6.66 |
