# Supplementary material for: Integration of genetic, genomic and transcriptomic information identifies putative regulators of adventitious root formation in Populus
Source: BMC Plant Biol. 2016 Mar 16;16:66. doi: 10.1186/s12870-016-0753-0 (PMC4793515; doi:10.1186/s12870-016-0753-0)
Supplement: Additional file 6: — Differentially regulated genes between time points. Number of genes differentially expressed when contrasting consecutive time points. (DOCX 44 kb) [file 12870_2016_753_MOESM6_ESM.docx]

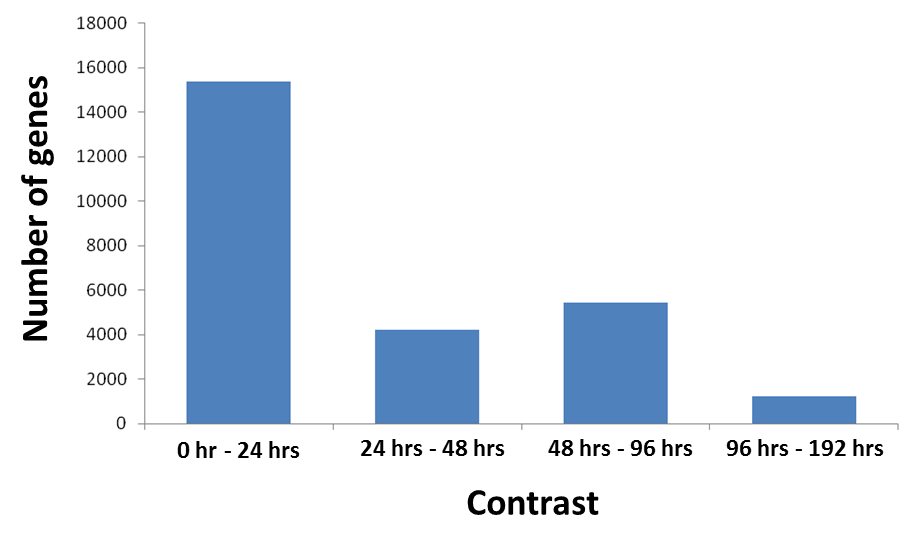


**Additional file 6.** Number of genes differentially expressed when contrasting consecutive time points.
