# Supplementary material for: Integration of genetic, genomic and transcriptomic information identifies putative regulators of adventitious root formation in Populus
Source: BMC Plant Biol. 2016 Mar 16;16:66. doi: 10.1186/s12870-016-0753-0 (PMC4793515; doi:10.1186/s12870-016-0753-0)
Supplement: Additional file 8: — Clustering the difference in transcriptome response of PtQTL and PdQTL genotypes. Modulated Modularity Clustering of genes displaying a similar pattern of expression differences between genotypes from the PtQTL and PdQTL categories, at all time points. (DOCX 25 kb) [file 12870_2016_753_MOESM8_ESM.docx]

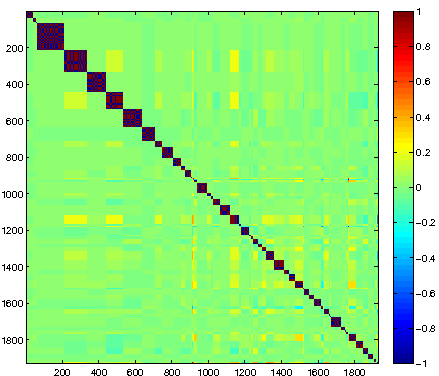


**Additional file 8.** Correlation matrix of the signal difference between genotypes in the *Pt*QTL and *Pd*QTL categories estimated for 1929 genes identified as differentially expressed (FDR<5%) between the two categories in at least one time point.
